# Cancer Stem Cells and Epithelial Ovarian Cancer

**DOI:** 10.1155/2010/105269

**Published:** 2011-01-24

**Authors:** Sheetal Dyall, Simon A. Gayther, Dimitra Dafou

**Affiliations:** ^1^Gynaecological Cancer Research Laboratory, EGA UCL Institute for Women's Health, London WC1E 6DD, UK; ^2^King's College School of Medicine, Guy's Hospital, London SE1 9RT, UK

## Abstract

The cancer stem cell hypothesis is becoming more widely accepted as a model for carcinogenesis. Tumours are heterogeneous both at the molecular and cellular level, containing a small population of cells that possess highly tumourigenic “stem-cell” properties. Cancer stem cells (CSCs), or tumour-initiating cells, have the ability to self-renew, generate xenografts reminiscent of the primary tumour that they were derived from, and are chemoresistant. The characterisation of the CSC population within a tumour that drives its growth could provide novel target therapeutics against these cells specifically, eradicating the cancer completely. There have been several reports describing the isolation of putative cancer stem cell populations in several cancers; however, no defined set of markers has been identified that conclusively characterises “stem-like” cancer cells. This paper highlights the current experimental approaches that have been used in the field and discusses their limitations, with specific emphasis on the identification and characterisation of the CSC population in epithelial ovarian cancer.

## 1. Introduction

Ovarian cancer (OC) is the sixth most lethal malignancy in women in the western world. Over 90% of malignant tumours are epithelial. It has been hypothesised that tumours can arise either from a single layer of cells covering the ovary (the ovarian surface epithelium or OSE) or from the epithelial lining of the fimbrial end of the fallopian tube [1]. The aetiology of OC remains poorly understood. One proposed model is the incessant ovulation hypothesis, which postulates that continuous rupture of the OSE during ovulation and subsequent cell proliferation leading to repair make OSE cells more susceptible to malignant transformation.

Approximately 70% of patients diagnosed with ovarian cancer have advanced stage disease, partly because symptoms are vague and can be confused with gastrointestinal complaints (e.g., bloating, constipation and mild abdominal pain) [2, 3]. Despite improvements in debulking surgery and initial good responses to platinum-based chemotherapies, survival rates for the disease remain poor due to the development of chemoresistant disease, and less than 60% of cases survive more than 5 years. Thus, the identification of molecular markers that target chemoresistance may represent suitable targets for new therapeutic approaches for epithelial ovarian cancers (EOC).

The mechanisms underlying chemoresistance in cancer are not clear. One hypothesis is that cancers are driven by a subset of highly tumourigenic cells with stem cell properties within the tumour, cancer stem cells (CSCs). The term CSC is not meant to suggest that these cells have any association with adult tissue stem cells; more recently, the term cancer or tumour initiating cells (CIC or TIC) has come to be thought of as more appropriate. As the term CSC is most commonly used in the literature to describe these cells, for the purpose of this paper, tumourigenic cancer cells with stem cell properties will be referred to as CSC. According to this model, only the CSCs, but not the remaining cells in the tumour, can propagate tumourigenesis. CSCs have been implicated in tumour initiation, progression, metastasis, and drug resistance.

## 2. Cancer Stem Cells

A recent AACR workshop defined the CSC as a malignant cancer cell with a stem cell phenotype [4]. Whilst the CSC hypothesis does not specifically address the mechanisms of malignant transformation, it has been suggested that CSCs are the malignant counterparts of normal adult tissue stem cells, which, due to dysregulated signalling pathways, are unable to maintain stem cell homeostasis. As is the case with tissue stem cells, CSCs are thought to reside at the top of the lineage hierarchy and give rise to differentiated cells, which themselves have no potential for self-renewal, and therefore do not contribute significantly to tumour growth.

The idea that tissue stem cells are the underlying cells for carcinogenesis is attractive. Due to their long lifespan, stem cells remain in a tissue for longer periods of time compared to their differentiated progeny, thereby making them more likely to acquire transforming mutations. Additionally, it is generally accepted that stem cells are more resistant to apoptosis and DNA damage and are therefore more likely to survive any insults [5, 6]. Whilst being quiescent in normal tissue, stem cells are able to maintain the stem cell pool by undergoing asymmetric cell division during processes such as tissue damage. During this process, a stem cell divides asymmetrically to generate an identical daughter cell (i.e., another stem cell) that is committed to differentiation. Adult stem cell can give rise to a wide range of differentiated cells and it has been suggested that CSCs undergo asymmetric cell division to generate the different cell types within a tumour, thereby contributing to tumour heterogeneity often seen in many cancers. The stem cell pool is also tightly regulated by signalling pathways from the microenvironment of the stem cell niche, and several of these pathways, including Hedgehog [7], and Wnt [8], have been implicated in carcinogenesis. 

The CSC hypothesis suggests a paradigm shift in the understanding of carcinogenesis and tumour cell biology. This may have fundamental implications in therapeutic interventions, including an explanation for the development of chemoresistance. A role for CSCs in propagating and maintaining metastasis has been proposed [9, 10]. Many features of tumours can be explained by the inherent characteristics of stem cells; for example, tumours selfrenew and heterogeneity can be attributed to multilineage differentiation. Dontu et al. [11] have proposed that breast cancers may arise from mammary stem cells, or early progenitor cells. It has also been shown that hypermethylation at Polycomb promoter regions lock stem cells in a state of self-renewal, which can lead to aberrant clonal expansion, thus leading to cancer predisposition [12]. Even so, the contrasting hypothesis that the dedifferentiation of mature cells to a more pluripotent state as a potential mechanism for the development of stem cell-like features by cancer cells cannot be dismissed.

Cancer cells with stem cell properties have been identified in acute myeloid leukemia and several solid malignancies (Table 1). Whilst the fact that tissue stem cells are the target of transformation may be true for certain malignancies, this has yet to be proven as a universal mechanism of tumorigenesis. Consequently, the terms cancer and tumour-initiating cells are thought to be more appropriate as they define cells with tumour-initiating potential, without any reference to an origin from tissue stem cells. 

The first strong evidence for the existence of cancer stem cells in human tumours came from a study in acute myeloid leukemia [13], in which the population of CD34^+^CD38^−^ cells isolated and transplanted into SCID mice formed tumours that had similar features to human leukemia, whilst populations containing cells with different cell surface marker profiles did not. Since then, several studies have characterised the CSC in other types of tumours (Table 1). 

Classically, stem cells are defined by their two main characteristics: self-renewal and pluripotency. And so by inference, to be classified as a CSC, a cell must meet the following criteria: (i) the ability of self-renewal and differentiation into different cell types, (ii) the ability to propagate tumours *in vivo,* (iii) the expression of distinct markers that permit consistent isolation, and (iv) restriction to a minority of the cell population (although this latter is now debatable, due to recent evidence showing that the microenvironment of mice alters the tumour-forming efficiency of engrafted cells) [14]. 

Table 1 summarises the various techniques and experimental approaches that have been used to isolate the putative CSC component from primary tumours. The approaches used rely on the intrinsic and functional properties of stem cells. The most commonly used approach is the tumour sphere assay, which evaluates the ability of cells to grow as nonadherent spheroids under non-differentiating conditions, and cell surface antigen profile of subpopulations found within tumours. The dye exclusion assay enables the isolation of a side population of cells that extrude lipophilic dyes due to the overexpression of drug efflux pumps; the enzyme aldehyde dehydrogenase 1 is thought to be preferentially active in stem-like cells. The tumourigenicity of the putative CSC component is then verified by engraftment into immunosuppressed mice. The aim is to show that a minimal number of stem-like cells can give rise to tumours of the same histopathology as the original primary tumours that they have been isolated from.

### 2.1. Assaying Cancer Stem Cells

#### 2.1.1. The Tumour Sphere Assay

Whilst there are no established protocols for isolating pure populations of CSCs, the general aim of many studies has been to enrich for cell populations that show features that are characteristic of normal stem cells, primarily the ability to self-renew and differentiate into different lineages. The tumourigenicity of the isolated cell populations is then confirmed by assessing the tumour-forming potential of these cells in appropriate mice models. A commonly used approach for CSC enrichment is the tumour sphere assay, which evaluates the ability of cancer cells to grow as multicellular spheroids under non-differentiating and non-adherent conditions, a characteristic believed to be indicative of self-renewal.

In a typical tumour sphere assay, cells from a primary tumour or cancer cell line are dissociated into a single cell suspension and cultured in a serum-free growth factor-rich medium containing primarily epidermal growth factor (EGF) and fibroblast growth factor (FGF). Multicellular spheroids of enriched CSCs that form can undergo serial dissociation and passaging to further enrich for stem cell-like cells. 

Although this is one of the most commonly used techniques to evaluate self-renewal, the results from such assays can be misleading. The characteristic of self-renewal can only be inferred from the presence of tumour spheres in culture if it can be shown that each tumour sphere has arisen from a single cell under proliferative nonadherent conditions; yet, there are few published studies that have actually demonstrated this [38]. A detailed analysis of the methodologies used in many studies shows that starting cell densities in the tumour sphere assay are fairly high (e.g., 1000 cells/mL). These studies aim to utilise single cell suspensions, but the high cell densities do not preclude the possibility that the spheroids that form may have arisen from the aggregation of mixed cell population and therefore would not be clonal. For neurospheres, it has been shown that spheroids are clonal only when cells are plated at low density [39].

#### 2.1.2. Cell Surface Specific Antigen Profile

Several studies have prospectively isolated CSCs by looking for the presence of extracellular markers that are thought to be stem cell-specific. The markers most commonly used are CD133 and CD44. CD133, a penta-membrane glycoprotein, has been found to interact with cholesterol and may be involved in maintaining membrane topology and membrane lipid composition. Its expression has been shown to be restricted to plasma membrane protrusions in epithelial cells [40]. It is also thought to be a marker for neuroepithelial progenitor cells and in haematopoietic progenitor cells. CD133 was originally identified as a neural stem cell marker. It has been used to characterise CSCs in brain tumours [17] as well as several other cancers, including, prostate, colon, ovary, and pancreas (Table 1). In breast cancer, CSCs have been characterised as CD44^+^ and CD24^−^ [41]. The limitation of using cell surface marker expression to characterise CSCs is that the approach requires prior knowledge of cell surface markers that are expressed by the putative CSCs in the tissue of interest, and often the choice of markers is inferred from known expression of markers in normal adult stem cells. 

Whilst CD133 and CD44 are thought to be indicative of a CSC phenotype, it is not clear if they are universal markers for characterising CSCs derived from all types of tumours. Furthermore, the expression of CD133 and CD44 may not be restricted to the CSC population and may be present in early progenitor cells. Their functions are largely unknown, although there are reports implicating CD44 in metastasis and chemoresistance. Recent evidence suggests that CD133 is expressed in epithelial cells and may be involved in maintaining pluripotency [40]. 

Epithelial-to-mesenchymal transition (EMT) is also a feature of carcinogenesis, as cells acquire a more mesenchymal phenotype during neoplastic transformation. A link between CSC and EMT has been suggested, whereby transformed human mammary epithelial cells that have undergone EMT show a gain of the CSC phenotype [42].

#### 2.1.3. Hoechst 33342 Dye Exclusion Assay

Stem cells actively efflux lipophilic compounds such as the fluorescence dyes Hoechst 33342 and rhodamine from their cytoplasm, as they overexpress ATP-binding cassette (ABC) transporter proteins on their cell surface, thus contributing to the intrinsic resistance of stem cells to chemotherapy and radiotherapy. This characteristic has been implicated in multidrug resistance in cancers, as overexpression of multi-drug resistant proteins enables cancer cells to remove chemotherapeutic drugs from their cytoplasm. 

When stained with the fluorescent dyes, cells overexpressing the ABC transporter proteins will eliminate the dye from their cytoplasm, thus producing a lower signal than cells not overexpressing ABC proteins. By combining fluorescent staining with fluorescent-activated cell sorting (FACS) in a cell population, a side population of cells (SP) can be isolated that is enriched for stem “like” cells. As a control for the SP phenotype, cells are stained in the presence of ABC transporter inhibitors, which reverse the SP phenotype. The most commonly used inhibitor is verapamil, which targets P-glycoprotein encoded by the gene *MDR1*. A less frequently used inhibitor is fumitremorgin C, which specifically targets the BCRP transporter (encoded by *ABCG2*). Cells that retain Hoechst are thought to contain terminally-differentiated cells that are unable to self-renew; thus this is a marker commonly used for identifying the non-SP fraction of a cell population. 

SP analysis, which was originally used to identify haematopoietic stem cells from murine bone marrow [43], has been used to isolate putative CSCs in several studies in primary tumours and cell lines [19, 28, 29, 36, 44–50]. Reviewing the published literatures suggests that the percentage of SP cells that can be isolated from whole cell line populations can range from 0%–20%. However, in the majority of studies, the SP cells form a minority (<5%) of the bulk cell population, supporting the idea that CSCs, like stem cells, constitute a small proportion of cell cultures.

SP analysis provides a rapid method for isolating stem cell-like cells; it relies on the functional characteristic of stem cells and requires no prior knowledge of extracellular markers. Nevertheless, the use of Hoechst as a method of enriching for CSC has received criticism. It has been reported that Hoechst treatment reduces the clonogenicity of the breast cancer cell line MCF7 and the ovarian cancer cell line SKOV3 due to a loss in viability [51]. In this study, single cells from unsorted cell populations, when grown in serum-containing media under adherent conditions, generated a clone that would satisfy the criteria for self-renewal. Additionally, the same group reported that both SP and non-SP cells generated clones *in vitro* and non-SP cells were able to form both SP and non-SP cells, suggesting that non-SP cells can self-renew as well.

#### 2.1.4. Aldehyde Dehydrogenase Expression

More recently, it has been suggested that an elevated level of aldehyde dehydrogenase 1 (ALDH1) activity can select for stem-like cells in both normal and malignant tissues. ALDH1 is a detoxifying enzyme that is involved in the metabolism of vitamin A to retinoic acid in the liver. It has been suggested that retinoic acid signalling is a probable protective mechanism against oxidative damage in stem cells; recently, levels of ALDH1 have been used as CSC markers in the isolation of putative CSC, and are thought to be more specific to the stem cell phenotype [10, 52–55]. In the colon, it has been shown that the expressions of CD44 and CD133, which have previously been used to isolate CSC populations, are not restricted to the stem cell populations, which are located at the base of colonic crypt, but also to populations of rapidly proliferating cells located further up along the crypt. The expression of ALDH1, however, is more limited to the base of the crypt.

#### 2.1.5. In Vivo Tumourigenicity Assays

Following the identification of the putative CSC population, cells are engrafted into appropriate mice models to show that they are more tumourigenic than their non-CSC counterparts. This is currently believed to be the gold-standard in the field. Generally, a small number of CSCs and non-CSCs are transplanted into mice, and it is shown that CSCs have a higher tumour-forming capacity than non-CSCs.

## 3. Cancer Stem Cells and Ovarian Cancer

Several characteristics of CSCs have been implicated in chemoresistance and radiotherapy. Due to the overexpression of extracellular multi-drug transport proteins, CSCs are able to efflux chemotherapeutic drugs from their cytoplasm. Stem cells, and possibly CSCs, are inherently quiescent; however, current drugs target rapidly cycling cells. Thus, the hypothesis is that whilst the majority of cells in the bulk of the tumour are eliminated, CSCs evade chemotherapy and are able to reinitiate tumour development. 

There is still uncertainty relating to the identification of CSCs in ovarian cancer. Several papers have described the isolation of ovarian CSCs using different approaches. The first study to be published identified clones established from tumour ascites that were able to form anchorage-independent spheroids and were shown to express the stem cell markers Oct 3/4 and Nanog and the progenitor marker Nestin [32]. Using flow cytometry, Ferrandina et al. [34] reported that two isoforms of CD133 (isoforms 1 and 2) were both expressed in human ovarian tumours at a higher frequency than in normal ovaries and metastatic omental lesions [34]. Szotek et al. [50] used flow cytometry to isolate a side population of cells from genetically engineered mouse ovarian cancer cell lines that expressed the multi-drug transporter protein BCRP1 and were insensitive to doxorubicin, suggesting a possible link between CSCs and chemoresistance. They also isolated a similar, although much smaller, side population of cells from the human ovarian cancer cell lines IGROV-1, OVCAR3, and SKOV3, but these SP cells were not further characterised.

Two other studies have independently defined the ovarian cancer stem cell by evaluating the CD44^+^CD117^+^ [37] and CD133^+^ [31] phenotypes. The latter suggests an epigenetic regulation of the CD133 promoter. Additionally, using CD44, stem-like cells were enriched from patients' samples and were characterised by Myd88 expression and chemokine and cytokine production [30]. Despite the different profiles described for CSCs by these studies, both studies reported that the CSC phenotype was more resistant to platinum-based therapy, which again, supports the idea that CSCs may be responsible for chemoresistance. 

Generally, these studies highlight the lack of consensus about the molecular characteristics of ovarian CSCs. It is likely that the expression of markers overlaps, and both CD133 and CD44 characterise the ovarian CSC. Alternatively, there may be more than one population of cells with stem cell properties in ovarian cancers. The study by Bapat et al. [32] postulated that stem cells are the target of transformation in ovarian cancer, due to the fact that a few of the clones isolated from ovarian cancer ascites spontaneously immortalised in culture, suggesting a model for disease development.

## 4. Issues with the Current Experimental Approaches

As described above, the experimental approaches used to identify and characterise populations of putative CSC can be classed in three major categories: (1) their isolation based on the expression of extracellular markers, (2) their growth in tumour sphere assays under non-differentiating conditions, and (3) dye exclusion due to the overexpression of drug efflux pumps. Additionally, chemotherapeutic treatment is thought to enrich for CSCs; thus, when exposed to chemotherapy drugs, CSCs are selected for [56]. It is not clear whether chemoresistance is an inherent feature of CSCs or whether they acquire this property throughout tumorigenesis. 

The most common approach that has been used to isolate CSCs is to select for cells by the expression of extracellular markers that may be associated with stemness. However, the rationale underlying the selection of markers used for this purpose is not always apparent. Although CD44 and CD133 are the two most commonly used markers, it is unclear whether or not they are ubiquitous. CD133 has been extensively used to isolate CSCs in cancers of the brain [18], prostate [57], pancreas [58], and ovary [31, 34]; but whilst it has been successfully used to isolate brain tumour stem cells, the use of CD133 in defining CSCs in colon and ovarian cancer has been more controversial. In the study by Ferrandina et al., CD133 was suggested to be a marker for ovarian cancer and was also used as an ovarian CSC marker in the study by Baba et al. [31]. Zhang et al. on the other hand have suggested that CD44^+^CD117^+^ phenotype is indicative of the ovarian CSC phenotype. In all of these studies, the characteristics of the stem cell phenotype were confirmed by *in vivo* models and spheroid forming assays. It is likely that both CD133 and CD44 are markers for stem cell-like cells in epithelial OCs but there are no existing studies that have compared the expression of CD44 and CD133 in EOCs. It is possible that both markers are expressed by the same population of ovarian CSC; alternatively, they may be specific to different populations of CSCs, or there may be other as yet unidentified markers that are ubiquitous markers for ovarian CSCs. Several studies have shown that ovarian cancers are extremely heterogeneous, and this may suggest that different populations of CSC can be responsible for tumourigenicity in different histological subtypes. 

Another issue raised by the published literature is that caution needs to be applied when interpreting the results from tumour sphere assays. It is only possible to infer self-renewal if it has conclusively been shown that multicellular spheroids arise from single cells. Furthermore, it is important to show that single cells isolated from such spheroids can themselves generate spheroids through serial passaging. 

Despite the amount of literature on CSCs, it is still not clear as to what constitutes a universal CSC-specific profile. The definition of the CSC hypothesis is constantly evolving to specifically address the mechanism of neoplastic transformation in different tumour types. It is becoming increasingly evident that there may be more than one population of CSC within a tumour. In addition, the cell of origin of CSCs is not evident, as CSCs can arise from either stem cells or progenitor cells. To be a true CSC, all the established criteria that characterise the stem cell properties of a cancer cell need to be satisfied.

## 5. Concluding Remarks

Ovarian cancer is a heterogeneous disease with histologically defined subtypes, and it is highly probable that CSCs are involved in tumour development. Despite the number of studies attempting to isolate ovarian CSCs, a well-characterised profile has not been established. It is essential that further studies are carefully designed to address all the functional characteristics of stem cells covering: (i) self-renewal, where a single CSC is shown to form multicellular spheroid *in vitro* and (ii) multilineage differentiation such that only the putative CSC would divide asymmetrically to generate several cell types. The tumourigenicity of these cells then needs to be validated in an appropriate *in vivo* model that is permissible to tumour formation. The development of chemoresistant disease represents a major hindrance to successful ovarian cancer treatment, and the identification of a molecular profile of ovarian CSC may aid to the development of more effective targeted therapy.

## Figures and Tables

**Table 1:** Studies that have attempted to characterise the cancer stem cell (CSC) component in the cancers are listed. Only the most relevant studies are referenced. The different experimental techniques used in the referenced studies are reviewed; this highlights the lack of consensus in the approach that can be best used to identify stem-like cells in cancers.

Cancer type	Cell surface antigen profile^a^	*In vitro* characterization				*In vivo* characterisation	References
		Dye exclusion assay^b^	Tumour sphere assay^c^	Clonality assays^d^	ALDH assay^e^		
Leukaemia	CD34^+^/CD38^−^	H 33342 (5 *μ*g/mL) 2 hours, 37°C; Inhibitor KO143 (200 nM)	SFM	Colony forming unit assay: number of colonies from leukemic SP and haematopoetic SP compared per 10^6^ cells plated	Not done	SCID	[15, 16]
			20 ng/mL Interleukin-3				
			100 ng/mL stem cell factor				
Brain	CD133^+^	H 33342 (5 *μ*g/mL) 90 minutes at 37°C Inhibitors Verapamil (50 *μ*M) or reserpine (100 *μ*M).	SFM	Primary spheres dissociated and plated at cell dilutions from 200 cells/well to 1 cell.	Not done	NOD-SCID	[17, 18]
			20 ng/mL EGF,				
			20 ng/mL bFGF				
			10 ng/mL LIF				
			60 *μ*g/mL *N*-acetylcysteine				
Breast	CD44^+^CD24^[-]/low^ OCT4^+^ CD133^+^	H33342 (5 *μ*g/mL) for 90 min, 37°C Inhibitor FTC 10 *μ*M	SFM	Single cells (1 cells per well in 96-well plate)	ALDEFLUOR-positive	NOD-SCID	[19, 20]
			10 ng/mL bFGF				
			20 ng/mL EGF				
			5 *μ*g/mL insulin				
			0.4% BSA				
Colon	CD133^+^	H33342 (5 *μ*g/mL) for 1.5–2 hours, 37°C, Inhibitor verapamil at 50 *μ*M for 10 minutes at room temperature before adding H33342	SFM	Single-cell derived tumour spheres (unsorted and CD133^+^, either spheroid-derived or primary)	Not done	NOD-SCID	[21, 22]
			0.6% glucose,				
			9.6 *μ*g/mL putrescine,				
			6.3 ng/mL progesterone				
			ITS				
			20 ng/mL EGF				
			10 ng/mL bFGF				
Endometrial	—	H33342 (5 *μ*g/mL) for 90 minutes, 37°C Inhibitor verapamil (50 *μ*M)	—	—	Not done	NOD-SCID	[23]
Head and neck	CD44^+^	H33342 5 *μ*g/mL at varying time points, 37°C; Inhibitor reserpine (5 *μ*g/mL)	SCM	Single cells GFP^+^ and GFP^−^	Not done	NOD-SCID	[24–26]
Pancreas	CD133^+^	—	SFM	1 cell/well in SFM by limiting dilution	Not done	Nude mice	[27]
			10 ng/mL FGF				
			20 ng/mL EGF				
			5 *μ*g/mL insulin				
			ITS				
			0.4% BSA				
Prostate	CD44^+^, *α*_2_ *β*_1_^hi^, CD133^+^	H33342 (5 *μ*M) for 90 minutes, 37°C; Inhibitor verapamil (50 *μ*M)	SFM	Single cells were plated at 1,000/mL on a plate coated with 0.5% agar	Not done	SCID	[9, 28, 29]
			B27				
			10 ng/mL EGF				
			10 ng/mL bFGF				
Ovary	CD133^+^ CD44^+^CD117^+^	H33342 (5 *μ*M) for 90 minutes, 37°C; Inhibitor verapamil (50 *μ*M)	SFM	—	Not done	BALB/c-nu/nu	[30–37]
			5 *μ*g/mL insulin				
			20 ng/mL EGF				
			10 ng/mL bFGF				
			0.4% BSA				

^a^Cell surface antigen profile expression by flow cytometry and immunohistochemistry techniques

^b^Dye Exclusion flow cytometry assays following treatment with Hoechst, coupled with ABC multidrug transporter inhibitors to isolate side population cells

^c^Sphere formation assays for multicellular spheroid forming ability under nonadherent conditions, which is characteristic of stem cells

^d^Clonality assays measure the ability of single cells to generate spheroids/clones

^e^Aldehyde Dehydrogenase assays measure the levels of ALDH1 in cells

Soft agar assay measures anchorage-independent growth due to loss of contact-inhibition and is an indicator of tumourigenicity.

EGF: Epidermal growth factor

bFGF: Basic fibroblast growth factor

LIF: Leukemia inhibitory factor

NSF: Neuronal survival factor

BPE: Bovine pituitary extract

KO143: Inhibitor of breast cancer resistant protein (BRCP)

SFM: Serum-free media

SCM: Serum-containing media.
